# Ubiquity and Diversity of Heterotrophic Bacterial *nasA* Genes in Diverse Marine Environments

**DOI:** 10.1371/journal.pone.0117473

**Published:** 2015-02-03

**Authors:** Xuexia Jiang, Hongyue Dang, Nianzhi Jiao

**Affiliations:** 1 State Key Laboratory of Marine Environmental Science, Xiamen University, Xiamen 361102, China; 2 Institute of Marine Microbes and Ecospheres, Xiamen University, Xiamen 361102, China; CAS, CHINA

## Abstract

Nitrate uptake by heterotrophic bacteria plays an important role in marine N cycling. However, few studies have investigated the diversity of environmental nitrate assimilating bacteria (NAB). In this study, the diversity and biogeographical distribution of NAB in several global oceans and particularly in the western Pacific marginal seas were investigated using both cultivation and culture-independent molecular approaches. Phylogenetic analyses based on 16S rRNA and nasA (encoding the large subunit of the assimilatory nitrate reductase) gene sequences indicated that the cultivable NAB in South China Sea belonged to the α-*Proteobacteria*, γ-*Proteobacteria* and CFB (*Cytophaga-Flavobacteria-Bacteroides*) bacterial groups. In all the environmental samples of the present study, α-*Proteobacteria*, γ-*Proteobacteria* and *Bacteroidetes* were found to be the dominant *nasA*-harboring bacteria. Almost all of the α-*Proteobacteria* OTUs were classified into three *Roseobacter*-like groups (I to III). Clone library analysis revealed previously underestimated *nasA* diversity; e.g. the *nasA* gene sequences affiliated with β-*Proteobacteria*, ε-*Proteobacteria* and *Lentisphaerae* were observed in the field investigation for the first time, to the best of our knowledge. The geographical and vertical distributions of seawater *nasA*-harboring bacteria indicated that NAB were highly diverse and ubiquitously distributed in the studied marginal seas and world oceans. Niche adaptation and separation and/or limited dispersal might mediate the NAB composition and community structure in different water bodies. In the shallow-water Kueishantao hydrothermal vent environment, chemolithoautotrophic sulfur-oxidizing bacteria were the primary NAB, indicating a unique nitrate-assimilating community in this extreme environment. In the coastal water of the East China Sea, the relative abundance of *Alteromonas* and *Roseobacter*-like *nasA* gene sequences responded closely to algal blooms, indicating that NAB may be active participants contributing to the bloom dynamics. Our statistical results suggested that salinity, temperature and nitrate may be some of the key environmental factors controlling the composition and dynamics of the marine NAB communities.

## Introduction

Nitrate is inarguably one of the most important nutrients in the ocean, which usually constitutes the major limiting factor controlling the productivity of many oligotrophic regions of the world oceans [1,2]. Microorganisms play important roles in marine nitrogen cycling, in which environmental nitrate is produced via nitrification and removed via denitrification, dissimilatory nitrate reduction to ammonium and assimilatory nitrate reduction [3]. Comparing to the other two nitrate removal processes, assimilatory nitrate reduction has obtained the least attention in research and thus is least understood about its biogeochemical roles and the identities and diversity of the responsible microorganisms [4]. Competition for nitrate between assimilatory nitrate reducing bacteria (also called nitrate assimilating bacteria, NAB) and phytoplankton may affect marine productivity and especially the new production in specific regions of the ocean [5,6] or even on a global scale [7].

Nitrate is one of the major nutrients discharged by rivers into estuarine and coastal environments. Excessive nitrate may stimulate the growth of specific phytoplankton, causing the formation of harmful algal blooms (HABs) [8–10]. NAB may help in HAB prevention or termination via their competition against phytoplankton for nitrate, especially in aquatic environments rich in readily utilizable organic carbon- [11–15]. However, very little is known about the NAB community composition, diversity and dynamics in such an episodic event of HAB [16].

Microbial nitrate assimilation requires assimilatory nitrate reductase, which catalyses nitrate reduction to nitrite. The *nasA* gene, which encodes the large subunit of the assimilatory nitrate reductase of heterotrophic bacteria, has been used as a specific functional biomarker to study the diversity of NAB in several environments, including the South Atlantic Bight, Barents Sea, North Pacific Gyre [16,17], northern South China Sea [18] and a seagrass bed in the Tampa Bay [19]. These investigations consistently identified *γ-Proteobacteria* as the predominant *nasA*-harboring bacteria. A study found that the relative abundance of environmental *Marinobacter nasA* gene was positively correlated with NO_3_^-^ concentration, implying the active role of the *γ-Proteobacteria* in nitrate assimilation [16]. In line with these major molecular-based findings, a very small number of NAB have been isolated from marine waters and the majority of these isolates are indeed affiliated with the γ-*Proteobacteria* [17,18]. However, genomic studies indicated that many other groups of bacteria harbor the *nasA* gene [20]. The diversity of marine NAB has not yet been fully discovered and further investigations are necessary.

Although NAB may be an important component of the marine ecosystem, they are currently not well studied and fully understood, especially pertaining to the fundamental ecology of their diversity and community structure on the global ocean scale and their potential ecophysiological functions and biogeochemical roles, particularly during marine catastrophic events or conditions such as the occurrences of HABs. In order to assess the diversity of the marine heterotrophic NAB and to obtain a broader overview of their biogeographical distribution in the world oceans, the *nasA* gene was employed to investigate the heterotrophic NAB community composition in different water depths (epipelagic, mesopelagic and bathypelagic zones) of the South China Sea and Indian ocean, and in coastal water, shelf margin and open ocean of the Pacific Ocean. Representative NAB were also isolated from several sampling stations and depths of the South China Sea to verify certain key results obtained using the *nasA* gene-based molecular approaches. In addition, the *nasA*-harboring bacterial communities associated with HABs were investigated, in order to obtain an understanding of the heterotrophic NAB community characteristics and dynamics pertaining to HAB events in the East China Sea.

## Materials and Methods

### Study areas and sample collection

The sampling sites in this study were located in the following areas: the East China Sea (ECS), the South China Sea (SCS), the western Pacific Ocean (WPO), and the Atlantic Ocean (S1 Table). No specific permissions were required for these locations and the field studies did not involve endangered or protected species. The ECS is characterized as an extensive continental shelf where excessive anthropogenic nutrient inputs via the Changjiang River have affected the coastal water quality and ecosystems [21,22]. Harmful algal blooms happened in the Zhejiang Province coastal area of the ECS in 2005; samples were collected in bloom-tracking cruises [23]. The SCS is characterized by its deep basin reaching 4700 m in depth and by its wide continental shelves to the northwest and south with voluminous runoff from several large rivers such as the Pearl River and the Mekong River [24]. The western Pacific Warm Pool, which contains surface seawater with high chlorophyll and temperature [25], and the Atlantic Ocean are typical oligotrophic open oceanic environments. The Kueishantao hydrothermal field, close to the northeastern Taiwan Island, is a shallow-water hydrothermal system, representing an extreme marine environment [26].

Seawater samples were collected from 7 cruises (S1 Table) using Niskin bottles. Two to three liters of seawater was filtered through 0.22-μm-pore-size polycarbonate filters (Millipore, Bedford, MA) with a vacuum of less than 0.03 MPa. The filters with captured microbes were immediately frozen and stored at -20ºC until further analysis.

### DNA extraction, PCR amplification, clone library construction and sequencing

DNA was extracted using the UltraClean Soil DNA kit (MoBio, San Diego, CA) according to the manufacturer protocol as previously described [27]. Primers *nasA*964 (5’-CAR CCN AAY GCN ATG GG-3’) and *nasA*1735 (5’-ATN GTR TGC CAY TGR TC-3’) were used to amplify the *nasA* genes from environmental DNA [17]. Although the forward primer *nasA*964 targets the *nasA*/*narB* gene sequences of both heterotrophic bacteria and *Cyanobacteria*, the reverse primer *nasA*1735 targets specifically the heterotrophic bacterial *nasA* gene sequences. Reaction mixtures (20 μl) contained 2 μl 10× LA PCR Buffer (Mg^2+^ plus), 150 μM deoxynucleoside triphosphates (dNTPs), 0.5 μM each primer, 0.5 U LA *Taq* polymerase (TaKaRa, Dalian, China) and 1 μl template DNA. Three independent amplifications were carried out in a T3 thermocycler (Biometra, Germany). The cycling program was carried out according to Cai and Jiao (2008), and PCR products were gel-purified, ligated into pMD18-T vectors (TaKaRa, Dalian, China), and transformed into *Escherichia coli* DH5a competent cells (TaKaRa, Dalian, China). Positive clones were randomly chosen and screened for inserts via colony PCR with M13 primers for the vector. Fifty to ninety clones were randomly selected for sequencing in each clone library (Invitrogen, Shanghai, China).

### Isolation and phylogenetic analysis of NAB

Seawater samples (50 μL each) collected from SCS were spread on NDGA medium plates supplemented with 10 mM NaNO_3_ and incubated for 2 days at 28°C [28]. Marine sediment samples were diluted with sterile artificial seawater and NAB cultivations were performed following the above-described seawater method. Colonies were picked randomly and streaked on new NDGA medium plates supplemented with 10 mM NaNO_3_. For DNA extraction, colonies from bacterial isolates were cultured in 3 mL NDG liquid medium overnight at 28°C. The universal PCR primers 27F (5′-AGA GTT TGA TCC TGG CTC AG-3′) and 1492R (5′-GGT TAC CTT GTT ACG ACT T-3′) were used to amplify the bacterial 16S rRNA gene sequences from genomic DNA extracted from NAB isolates [29]. The *nasA* functional gene was also amplified to confirm the genetic potential for nitrate assimilation in the isolates.

### Phylogenetic and statistical analyses

The partial gene sequences of *nasA* obtained in the current study were translated into protein sequences using the EditSeq program (Lasergene software package) [30]. Multiple sequence alignments were performed using the CLUSTALX 1.81 program [31]. The neighbor-joining (N-J) phylogenetic trees were inferred using software Mega 4.0 [32]. Maximum likelihood (ML) phylogenetic trees were constructed with RAxML [33] and edited with the online tool iTOL [34,35].

Rarefaction analysis and coverage estimation of the constructed clone libraries were conducted using the DOTUR program [36]. Community classification of the *nasA*-harboring bacterial assemblages was performed using the Jackknife environment clustering and principal coordinates analysis (PCoA) with the Fast UniFrac program [37]. The ANOSIM analysis of clone libraries used by software PAST (http://folk.uio.no/ohammer/past). Correlations of the *nasA*-harboring bacterial assemblages with environment factors were explored using the canonical correspondence analysis (CCA) with software CANOCO (version 4.5, Microcomputer Power, Ithaca, NY) following previously published procedures [38,39].

### Nucleotide sequence accession numbers

The sequences obtained in this study have been deposited in the GenBank database under accession numbers JX533652 to JX533721, HQ734258 to HQ734695, HQ825100 to HQ825136, JQ627871 to JQ628106 and JQ628380 to JQ629401.

## Results

### Diversity of *nasA* genes based on phylogenetic analyses

**Fig 1:**
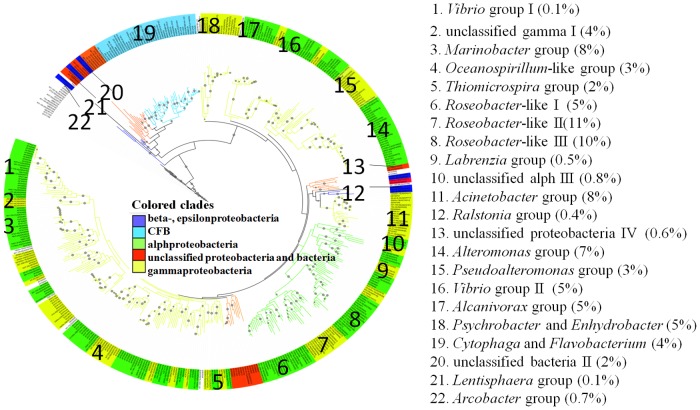
Maximum likelihood phylogenetic tree based on the *nasA* sequences. Nodes supported by bootstrap values >50% are indicated by grey circles. 52 *nasA* sequence groups were separated by different color ranges. The numbers on the graph represented the taxonomic *nasA* gene groups showing on the right. The percent of each group to all sequences is showed in the brackets.

A total of 1735 *nasA* gene sequences were obtained from all samples (S2 Table), resulting in 254 OTUs based on 95% protein sequence identity cutoff. The majority of the retrieved sequences may represent novel *nasA* gene sequences with only 36% of the total sequences exhibiting 90% or higher identity with the closest-match known sequences from the NCBI GenBank database. The *nasA* gene sequences were affiliated with 7 bacterial lineages (Fig. 1): α-, β-, γ-, ε-*Proteobacteria*, *Bacteroidetes*, Lentisphaerae, and unclassified bacteria. More than half (60%) of the sequences were related to γ-*Proteobacteria* and they were classified into 29 major groups. *Marinobacter* constituted the most abundant group (163 clones, 8 OTUs), and sequences in this group were present in all samples that were collected from different marine environments. The *nasA* gene sequences affiliated with the *Acinetobacter* group (153 clones) were assigned to 13 OTUs, mainly distributed in the coastal waters of the ECS. The *nasA* sequences affiliated with the *Alteromonas* group (138 clones, 13 OTUs) were prevalent in all sampling regions and at all depths. Sequences belonged to *Vibrio* (107 clones, 11 OTUs) were distributed in two independent phylogenetic groups. Sequences in the *Alcanivorax* group (92 clones, 9 OTUs) were widely distributed in the water column of the ECS and SCS. Sequences affiliated with *Psychrobacter* (89 clones, 8 OTUs) and *Enhydrobacter* (16 clones, 1 OTU) were distributed mainly in the ECS, SCS and WPO. Sixty-four *nasA* sequences were affiliated with the *Pseudoalteromonas* group. Sequences affiliated with the *Oceanospirillum*-like group (66 clones, 7 OTUs) were mainly distributed in the ECS surface water. The *Thiomicrospira* group (42 clones, 1 OTU) constituted the dominant *nasA*-harboring bacteria in the Kueishantao hydrothermal field. More than 260 *nasA* sequences were related to unidentified γ-*Proteobacteria*-affiliated sequences.

Thirty percent of the *nasA* gene sequences obtained in the current study were affiliated with the α-*Proteobacteria* (528 clones, 63 OTUs). More specifically, the majority (90%) of the α-*Proteobacteria* sequences were affiliated with the marine *Roseobacter*-like clade. *Roseobacter*-like I (105 clones, 12 OTUs) and *Roseobacter*-like II (223 clones, 28 OTUs) clusters were consisted of *nasA* gene sequences very distantly related to known sequences. Sequences in the *Roseobacter*-like I cluster that occurred mainly in ECS and SCS were distantly related to *nasA* gene sequences obtained from bacteria with fully sequenced genomes. Sequences in the *Roseobacter*-like II cluster were distributed mainly in the western Pacific Ocean and SCS. Most of the remaining α-*Proteobacteria nasA* sequences were related to unidentified α-*Proteobacteria*.

Eight *nasA* gene sequences from ECS were grouped with *Ralstonia solanacearum* in β-*Proteobacteria* and 2 sequences from Kueishantao hydrothermal field were grouped with *Arcobacter* in ε-*Proteobacteria*.

A large cluster containing 86 clones (22 OTUs) belonged to phylum *Bacteroidetes*, with half of the sequences (43 sequences, 9 OTUs) being affiliated with known bacteria in the *Cytophaga*-*Flavobacterium* group and 23 sequences (8 OTUs) being affiliated with unclassified *Flavobacteriaceae*. The remaining 20 sequences were affiliated with *Algoriphagus* (5 OTUs). Two sequences obtained from the ECS were affiliated with *Lentisphaera*. The remaining sequences were from unidentified bacteria.

### Spatial distribution of the marine *nasA*-harboring bacteria

**Fig 2:**
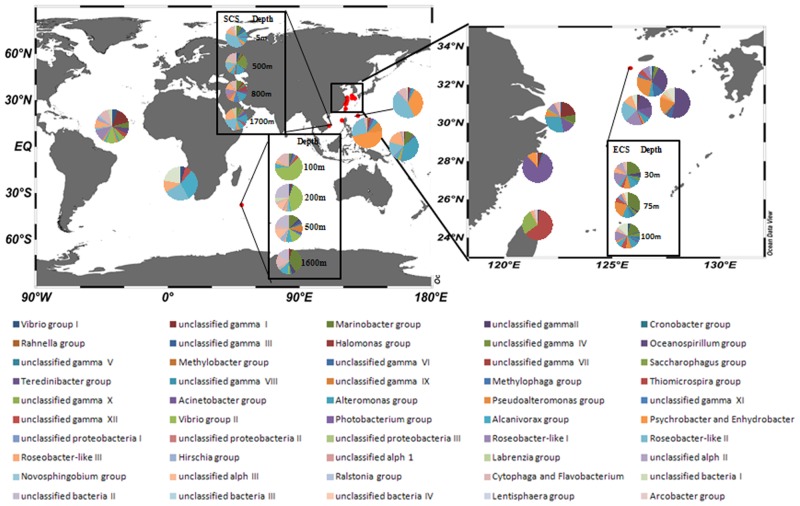
The map of sampling locations. Pie charts display the distribution of *nasA* gene groups within each sample with different NAB groups represented by different colors.

**Fig 3:**
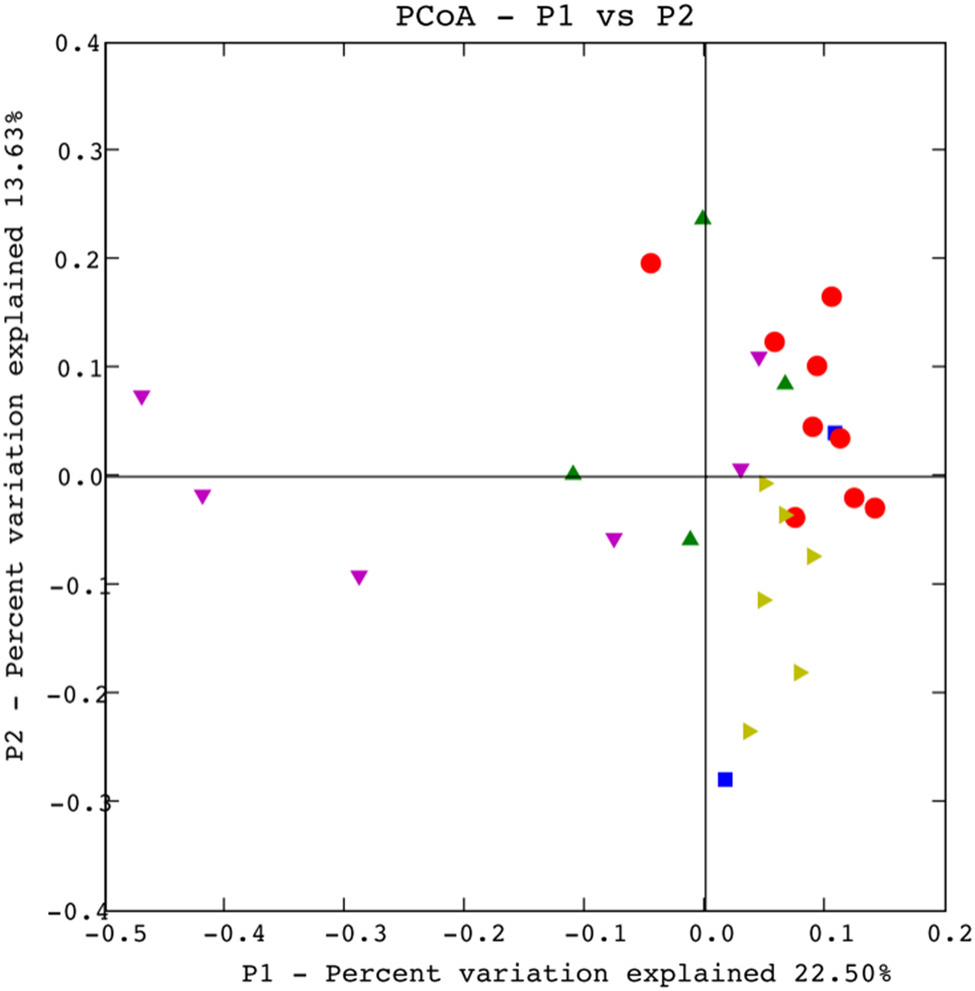
PCoA of the nitrate-assimilating communities with Fast UniFrac using the *nasA* sequences. WPO samples showed by purple triangle, Indian Ocean samples showed by bottle-green triangle, SCS samples showed by absinthe-green triangle, ECS samples showed by red circle and Atlantic samples showed by blue square.

The *nasA*-harboring bacterial assemblages showed obvious biogeographical distribution difference (Fig. 2). The existence of different ecotypes and the heterogeneous geographical distribution of the *nasA*-harboring bacterial assemblages were confirmed via Fast UniFrac PCoA analysis (Fig. 3). Clone libraries of surface water samples clustered into several groups. Samples from the Indian Ocean (unpublished data), western Pacific Ocean, ECS and SCS grouped separately. Higher *nasA* gene sequence diversity in the SCS and ECS than that in the Indian Ocean was supported by rarefaction curve and diversity index analyses (S1 Fig., S2 Table).

Regionally, our study showed that the *nasA* gene sequence diversity of the ECS coastal waters was lower and the composition of the *nasA* genotypes was unique (S2 Fig.). Most of the *nasA* sequences from the ECS coastal waters were affiliated with *Acinetobacter* and a specific unclassified γ-*Proteobacteria* group. The *nasA* gene sequences from ECS shelf and SCS open sea samples were more diverse and the *nasA*-harboring bacterial community composition was different from that of the coastal waters. Our clone library analysis showed higher *nasA* gene sequence diversity in the western Pacific Warm Pool than that in the western Pacific gyre. Almost all the subclusters of the *nasA* gene sequences of the western Pacific gyre samples can be found in the western Pacific Warm Pool. Besides, the *nasA*-harboring bacterial assemblage of the Kueishantao hydrothermal field was unique, with 63% sequences being affiliated with *Thiomicrospira*. These results showed that the *nasA*-harboring bacterial assemblages varied among different environments of the western Pacific marginal seas.

**Fig 4:**
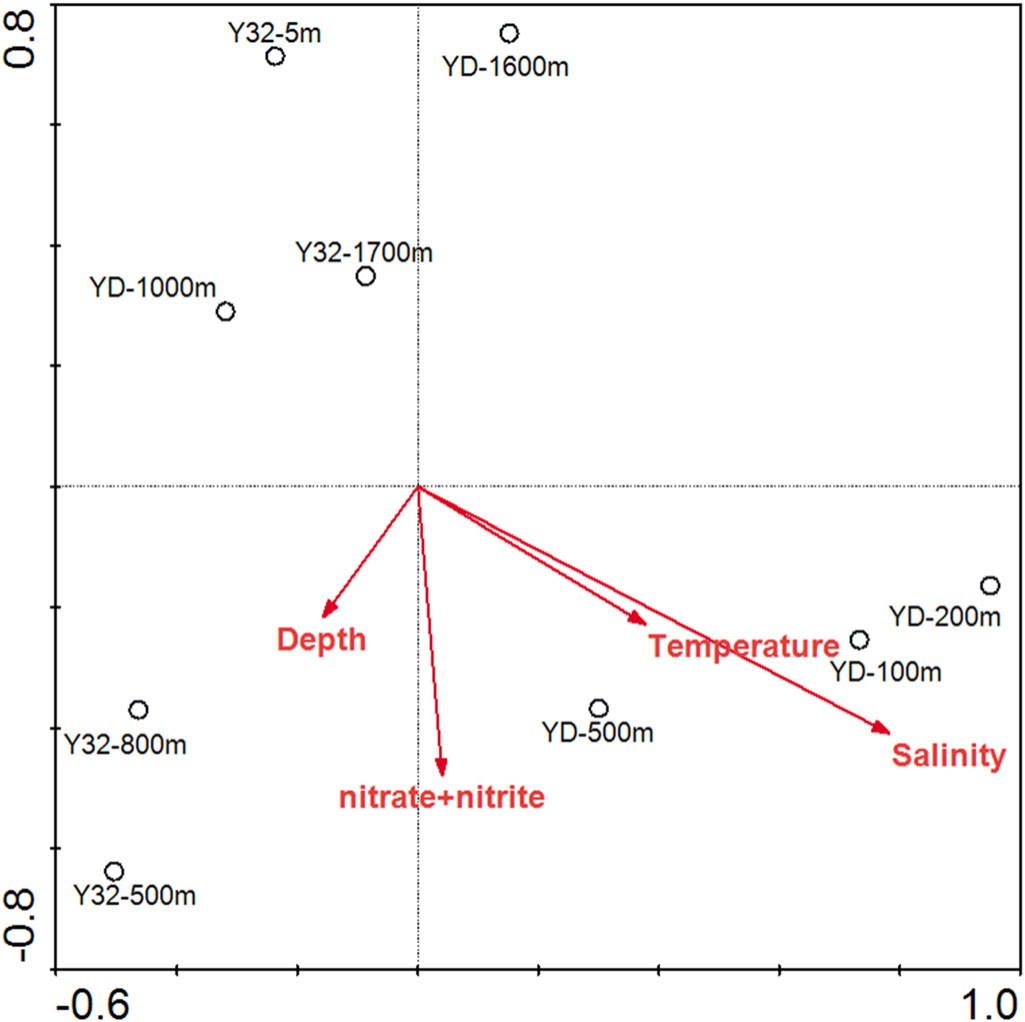
CCA ordination plots of the relationship between the environmental parameters with the NAB groups in the vertical dimension of Indian Ocean and South China Sea.

Samples from different water depths in the same station share high similarity of the clone library *nasA* gene sequence composition based on the clustering analysis (Fig. 3). To test the environmental factors shaping the community structure of *nasA*-containing bacteria in the vertical dimension, CCA analysis was performed (Fig. 4). Of all the environmental factors analyzed, salinity (*p* = 0.027) was identified as the most significant environmental factor.

### NAB community change in response to algal blooms

**Fig 5:**
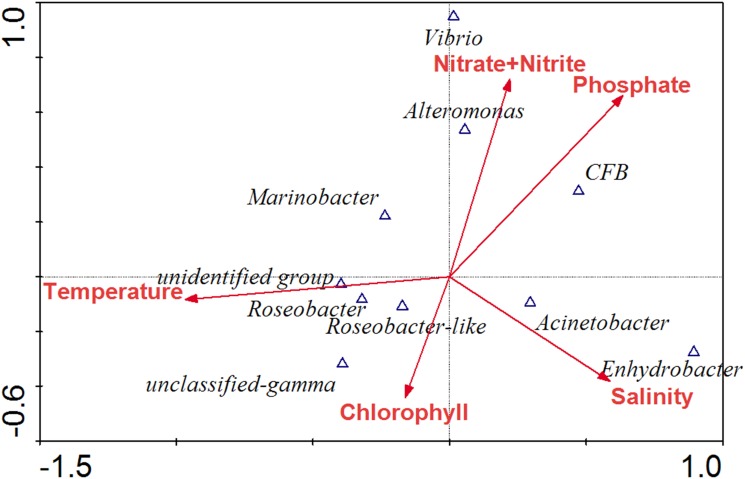
CCA ordination plots of the relationship between the environmental parameters with the NAB groups in the red tide-occurring area of ECS coast.

The *nasA*-harboring bacterial diversity of the bloom-occurring stations (RA01, ZD-24a and ZB09) was compared with the pre-bloom stations (RA01 and ZE-27) of the ECS. Our results showed that the *nasA*-harboring bacterial diversity increased at station RA01 when *Prorocentrum donghaiense* and *Karenia mikimotoi* blooms occurred simultaneously, while decreased at station ZD-24a when a *Noctiluca scintillans* bloom occurred. At station RA01, *nasA* gene sequence groups affiliated with *Alteromonas* and *Vibrio* disappeared, while *nasA* gene sequences affiliated with *Roseobacter*-like and unclassified γ- and β-*Proteobacteria* increased when blooms occurred. At the other bloom-occurring stations (RA01 and ZB09), *Roseobacter*-like *nasA* gene sequences also increased significantly. The CCA analysis showed that temperature (*p* = 0.001) and nitrate (*p* = 0.089) were identified as the most significant environmental factors in shaping the *nasA*-harboring bacterial assemblages in bloom area (Fig. 5). *Alteromonas* and *Vibrio* groups were positively correlated to nutrients, while the *Roseobacter*-like group was mainly influenced by temperature and seawater Chlorophyll content (Fig. 5).

### Isolates of NAB

**Fig 6:**
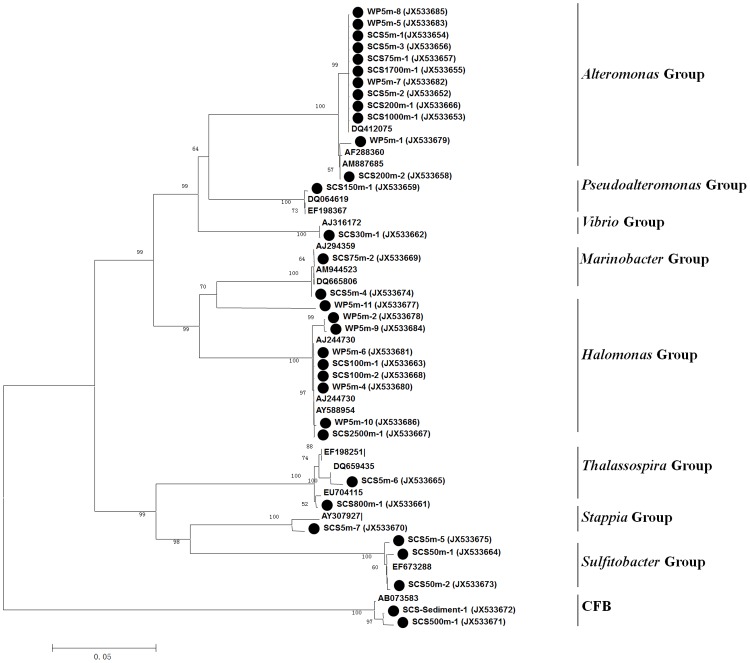
Phylogenetic analysis of cultivable *nasA*-harboring bacteria based on the 16S rRNA gene sequences.

Bacterial colonies growing on NDGA agar plates were diverse in color and morphology. After visual screening based on different colony morphology, color and size, 89 strains were isolated into pure cultures for further identification by phylogenetic analyses using the amplified 16S rRNA and *nasA* gene sequences (Fig. 6, S3 Fig.). Among them, 54 isolates were *nasA* gene positive. Isolates with identical or one-base-difference 16S rRNA gene sequences were considered as the same strain, and consequently 35 distinct strains were obtained (S3 Table). Cultivable NAB belonged to the α-, γ-*Proteobacteria* and CFB groups and were affiliated specifically with the genera *Alteromonas*, *Aeromonas*, *Pseudoalteromonas*, *Halomonas*, *Marinobacter*, *Vibrio*, *Acinetobacter*, *Sulfitobacter*, *Thalassospira*, *Stappia*, and *Maribacter*. Of these, the most abundantly cultivable NAB were *Alteromonas* (18 isolates in total 54 isolates). Most isolates in this group were identified with 99–100% sequence identity to the 16S rRNA gene sequences of known bacteria, though our isolates were isolated from different water depths of the SCS (S3 Fig.). The NAB isolates in the second most abundant group were affiliated with *Sulfitobacter* within the marine *Roseobacter* clade. Other α-*Proteobacteria* NAB, including strains affiliated with *Thalassospira* and *Stappia*, were additionally isolated. *Alteromonas* and *Sulfitobacter* were the predominant cultivable NAB, accounting for 30% and 20% of all the strains, respectively.

## Discussion

### Diversity of the *nasA*-harboring bacteria in the global oceans

In this study, *nasA*-harboring bacteria were found to be highly diverse and ubiquitously distributed in the studied marginal seas and world oceans. These bacteria belonged to α-, β-, γ-, ε-*Proteobacteria*, *Bacteroidetes*, Lentisphaerae and unclassified bacteria, suggesting that some of the marine NAB groups have not been recognized. The γ-*Proteobacteria* constituted the most abundant *nasA*-harboring bacteria in our gene clone libraries, consistent with results of previous studies [17,18]. The γ-*Proteobacteria* are well known for their adaptability and opportunism [45,46]. High requirement of nitrogenous nutrients may be a basic physiological characteristic that is required for the functioning of these bacteria [5]. One study showed that the growth of γ-*Proteobacteria* and *Bacteroidetes* was positively correlated with nitrate [46]. Consistent to our molecular study results, bacterial isolates related to γ-*Proteobacteria* were found to be the most diverse and abundant NAB group, of which *Alteromonas* were the most frequently recovered isolates. It has previously been found that *Alteromonas* were prevalent and biogeochemically important in carbon cycling in the ocean [47–49]. Marine γ-*Proteobacteria* and especially *Alteromonas* are also likely the major players contributing to the consumption and assimilation of environmental nitrate in the ocean.

The α-*Proteobacteria nasA* sequences were also especially rich in our gene clone libraries and they exhibited high divergence with known sequences (over 90% cloned sequences shared < 90% protein sequence identities with the closest-match GenBank sequences). The *nasA* gene sequences of the α-*Proteobacteria* lineage were dominant in the western Pacific Ocean. The result was in agreement with a previous analysis of 16S rRNA gene sequences that showed the dominance of α-*Proteobacteria* in the seawater bacterial community across the transect through the western Pacific Warm Pool [40]. High genetic diversity of *nasA* genes in the western Pacific Warm Pool could be related to the high chlorophyll content [25]. Although stable isotope probing experiments indicated the nitrate assimilation potential of marine α-*Proteobacteria* in the West Florida Shelf water [50], α-*Proteobacteria* NAB and their *nasA* gene sequences were seldom found or not dominant in most previous studies that employed either cultivation-based or *nasA*-based screening approaches (S4 Table) [16–19]. The results of our current study indicate that the *nasA*-harboring α-*Proteobacteria* may be another major group of NAB in the ocean, likely having been overlooked previously.

Half of our α-*Proteobacteria*-related *nasA* gene sequences were closely related to the *nasA* sequence of *Maritimibacter alkaliphilus* HTCC2654^T^, a bacterium affiliated within the marine *Roseobacter* clade originally isolated from the western Sargasso Sea [51]. The *nasA* genes have been found in the genomes of many *Roseobacter* clade bacteria (S5 Table). For example, the genome of *Pelagibaca bermudensis* HTCC2601 contains a complete assimilatory nitrate reduction pathway [52]. It has also been confirmed that the marine *Roseobacter* clade bacteria indeed assimilate nitrate in natural marine environments [50]. The importance of α-*Proteobacteria* NAB and especially their *Roseobacter* clade members was further verified by our cultivation-based research results (Fig. 6, S3 Fig.). The *Roseobacter* clade isolates constituted the second largest cultivable NAB group. The α-*Proteobacteria nasA* gene sequences were only occasionally found in a couple of previous studies [18,19]. Our study is probably the first report that diverse and abundant *Roseobacter* clade NAB were identified by both molecular and cultivation screening, to the best of our current knowledge. Many species in the marine *Roseobacter* clade are typical aerobic anoxygenic phototrophic bacteria (AAPB) [53,54]. Globally, AAPB represent a functionally important bacterial group and play a particular role in the ocean carbon cycle [55]. Our study indicates that they may also actively participate in marine nitrate consumption and assimilation. The ability to assimilate inorganic nitrogenous nutrients such as nitrate is consistent to the energy metabolic strategy of AAPB that utilize light energy to lower the expense of organic matter for ATP production [56].

In the present study, we tentatively found *Bacteroidetes* as important NAB. *Bacteroidetes* are abundant in coastal and nutrient-rich environments, being a major biopolymer-degrading bacterial groups. They possess diverse extracellular enzymes specific to the degradation of algal polysaccharides and they also harbor abundant carbohydrate-assimilation TonB-dependent transporters [57,58,59,60]. Algal polysaccharides typically are carbon-rich but nitrogen-poor organic matter. When *Bacteroidetes* utilize the carbon source derived from algal polysaccharides, they may need to take up extra sources of nitrogen such as nitrate. This putatively explains why *Bacteroidetes* may be important NAB in marine environment, and this hypothesis warrants further investigation.

### Spatial distribution pattern of NAB in different water columns

The *nasA*-harboring bacterial assemblages were quite different biogeographically in different environments of the oceans (Fig. 2, Fig. 3). The ANOSIM analysis showed the NAB communities were different among SCS, ECS and western Pacific Ocean (S6 Table). Our data, combined with previous data from other regions of the oceans, demonstrate niche adaptation-mediated and/or limited dispersal-mediated *nasA*-harboring bacterial community distribution in different water bodies of the world oceans.

Most of the *nasA* gene sequences and NAB isolates obtained in the current study were related to heterotrophic bacteria, as expected from the specificity of the reverse PCR primer [17]. However, in the extreme environment of the Kueishantao hydrothermal field, the *nasA*-harboring bacterial assemblage was very unique, with the majority of the *nasA* sequences being affiliated with *Thiomicrospira*. Genes encoding dissolved inorganic nitrogen transporters are present in the genome sequence of *Thiomicrospira crunogena* XCL-2 [41]. The gene clone library of the Kueishantao hydrothermal field sample also contained the *nasA* gene sequences affiliated with the ε-*Proteobacteria Arcobacter*. Both *Thiomicrospira* and *Arcobacter* can carry out sulfur oxidization [42,43]. There are massive sulfur deposits around the studied hydrothermal vent [44]. Thus, chemolithoautotrophic bacteria involved in sulfur metabolism are likely the primary *nasA*-harboring bacteria in this shallow-water hydrothermal vent environment. This first report for such an extreme marine environment revealed the existence of distinct *nasA*-harboring bacteria.

The *nasA*-harboring bacterial assemblages from surface seawater to abyssal depths were compared among the ECS, SCS and Indian Ocean. Phylogenetic analysis revealed difference in *nasA*-harboring bacterial community composition among the three environments. The SCS was characterized by high *nasA*-harboring bacterial diversity and abundant α-*Proteobacteria*-affiliated sequences (*Roseobacter*-like II, *Roseobacter*-like III and *Labrenzia* groups), ECS was dominated by *Oceanospirillum*-like, *Roseobacter*-like I and *Pseudoalteromonas* groups, while unclassified α-*Proteobacteria* III, unclassified bacteria II, *Roseobacter*-like III and *Vibrio* group II were the dominant *nasA*-harboring bacterial groups in the Indian Ocean. These comparative results revealed that niche separation and/or limited dispersal may cause the different *nasA*-harboring bacterial populations. For example, the *nasA*-harboring bacterial assemblages from the five depths of the Indian Ocean samples were grouped together and they were separated from the ECS and SCS seawater samples (Fig. 3).

As most of the *nasA* gene sequences obtained in the current study were affiliated with heterotrophic bacteria, it is reasonable to hypothesize that carbon availability or energy metabolism relying on other sources of energy such as light for AAPB may play an important role in shaping the *nasA*-harboring bacterial community structure. A recent study about nitrate reductase gene expression of fungi in agricultural soils suggested that energy supply by a carbon source was the major regulator [61]. Another study showed that high organic matter inputs had a significantly positive effect on nitrate assimilation by soil microbes [62]. The expression of nitrate reductase genes is linked to the availability of an energy source such as labile dissolved organic matter (DOM) or light [63]. Unfortunately, however, environmental factors related to the dissolved and particulate forms of aquatic organic matter were not measured in the current study. This hypothesis warrants further investigation.

### The responses of *nasA*-harboring bacteria to algal blooms

River export of nutrients, especially dissolved inorganic N and P, to China seas increased in the past years and could influence the diversity and complexity of the *nasA*-harboring bacterial assemblages. NAB may be a nitrate sink in the coastal seas, playing important roles in responding to the eutrophication events and maintaining the stability of the nutrient cycles. The increasing anthropogenic nutrient discharge into the sea could reduce the microbial carbon sink in the estuarine and near-shore waters [64]. The coastal region of the ECS is prone to algal blooms, especially in our study area. In the current study, we investigated the *nasA* gene sequence diversity in the coastal area of the ECS when algal blooms occurred. Our comparative results revealed different responses of *nasA*-harboring bacteria to algal blooms. The relative abundance of the *Roseobacter*-like *nasA* gene sequences increased dramatically when *Thalassiosira curviseriata* and *Skeletonema costatum* blooms occurred. The putative increase of *Roseobacter*-like *nasA* sequences in the bloom phase could be explained by the enhanced release of DOM from the bloom species. One study at the same station showed that the abundance of AAPB cells increased in the bloom phase, with organic material released from the blooming phytoplankton being identified as a key controlling factor [23]. The genomes of some AAPB strains, such as *Jannaschia* sp. strain CCS1 and *Roseovarius* sp. strain 217, contain the *nasA* genes [65]. Another study showed that the composition of the bacterial communities growing on the algal exudates differed markedly depending on the phytoplankton species [66]. The difference of the *nasA*-harboring bacterial populations in responding to algal bloom species suggested that algae-released DOM may play an important role in shaping the NAB community structure. On the other hand, NAB may play an important role in the bloom nitrate dynamics, as these bacteria may actively participate in the in situ nitrate assimilation process. How NAB may contribute to the algal bloom development or termination needs to be further investigated.

In conclusion, this study presents the first large-scale systematic survey of the biodiversity of NAB in several oceans and marginal seas using both *nasA* gene- and cultivation-based approaches. The *nasA*-harboring bacteria have adapted and distributed in various marine environments. We confirmed that many species of NAB in the α- and γ-*Proteobacteria* and in the CFB group of *Bacteroidetes* can be easily cultivated. We found that the marine *Roseobacter* and *Alteromonas* bacterial groups are especially important in nitrate assimilation and in responding to HAB events. We also found that several environmental factors may play important roles in mediating the *nasA*-harboring bacterial composition and community structure.

## Supporting Information

S1 Fig Rarefaction curves of *nasA* clone libraries in the vertical dimension (Y32 and Z95 represent SCS stations，601 represents ECS station，YD represents Indian Ocean station). (TIF)

S2 Fig Rarefaction curves of clone libraries in the surface water samples. (TIF)

S3 Fig Diversity of cultivable *nasA*-containing bacteria based on the *nasA* sequences (TIF)

S1 Table Cruise names and sampling time in this study. (XLSX)

S2 Table Clone libraries and environmental parameters at the sampling locations. (XLSX)

S3 Table Blast results of *nasA* gene and 16S rRNA of the isolated nitrate-assimilating bacteria with the nearest strains in the GenBank. (XLSX)

S4 Table Alphaproteobacterial nasA sequences information in published references. (XLSX)

S5 Table The *nasA*-containing microorganisms in the NCBI genomic database. (XLSX)

S6 Table The ANOSIM analysis of clone libraries from different oceanic environments. (XLSX)
