# Correction: Ubiquity and Diversity of Heterotrophic Bacterial *nasA* Genes in Diverse Marine Environments

**DOI:** 10.1371/journal.pone.0137817

**Published:** 2015-09-03

**Authors:** Xuexia Jiang, Hongyue Dang, Nianzhi Jiao

The following information is missing from the Funding section: State Oceanic Administration grant number 201105021 and China National Offshore Oil Corporation grants CNOOC-KJ 125 FZDXM 00 TJ 001–2014 and CNOOC-KJ 125 FZDXM 00 ZJ 001–2014.

The complete, correct Funding statement is: This work was supported by China MOST 973 grant 2013CB955700 (http://www.973.gov.cn/English/Index.aspx), NSFC grants 91028001, 91328209, and 91428308 (http://www.nsfc.gov.cn/publish/portal1), SOA grants 201105021 and GASI-03-01-02-05 (http://www.soa.gov.cn/index.html), and CNOOC grants CNOOC-KJ 125 FZDXM 00 TJ 001–2014 and CNOOC-KJ 125 FZDXM 00 ZJ 001–2014 (http://www.cnooc.com.cn/). The funders had no role in study design, data collection and analysis, decision to publish, or preparation of the manuscript.

## References

[1] Jiang X, Dang H, Jiao N (2015) Ubiquity and Diversity of Heterotrophic Bacterial *nasA* Genes in Diverse Marine Environments. PLoS ONE 10(2): e0117473 doi: 10.1371/journal.pone.0117473 25647610 10.1371/journal.pone.0117473 PMC4315400

