# Supplementary figures and images for: Ubiquity and Diversity of Heterotrophic Bacterial nasA Genes in Diverse Marine Environments
Source: PLoS One. 2015 Feb 3;10(2):e0117473. doi: 10.1371/journal.pone.0117473 (PMC4315400; doi:10.1371/journal.pone.0117473)

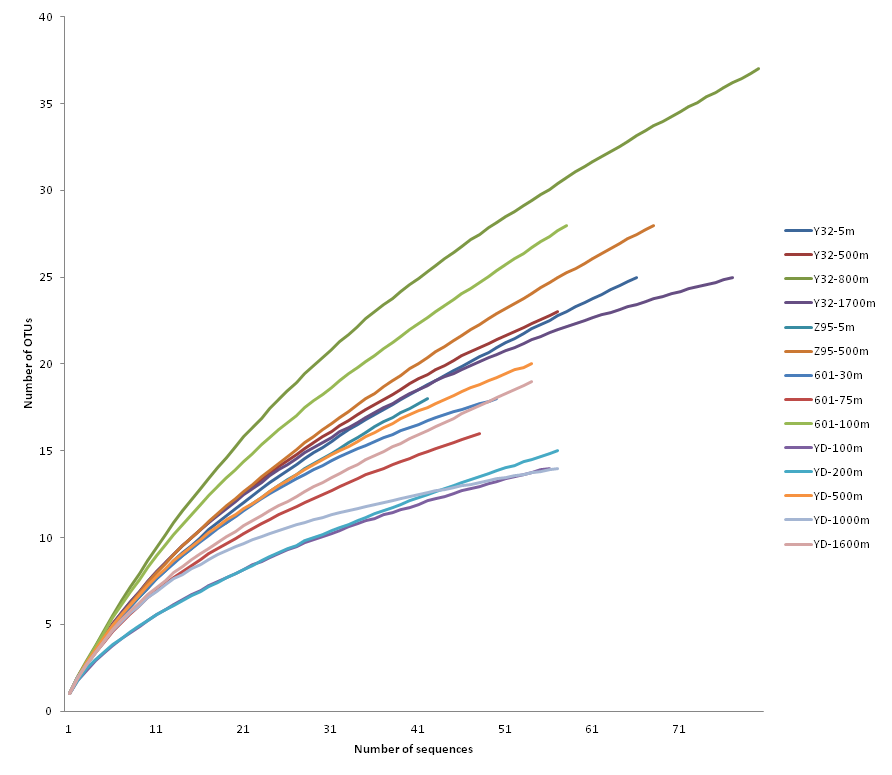

Supplement: S1 Fig — (TIF) [file pone.0117473.s001.tif]

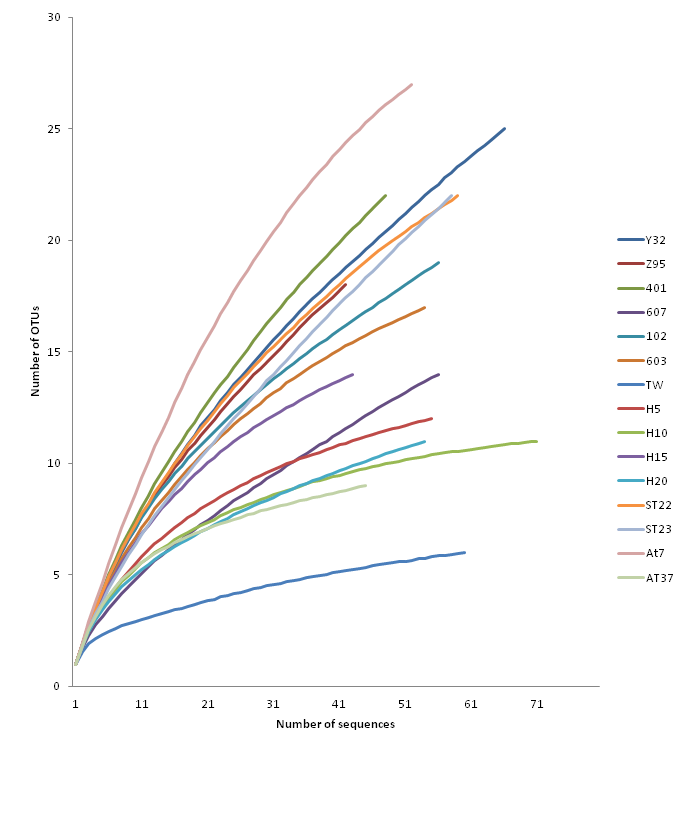

Supplement: S2 Fig — (TIF) [file pone.0117473.s002.tif]

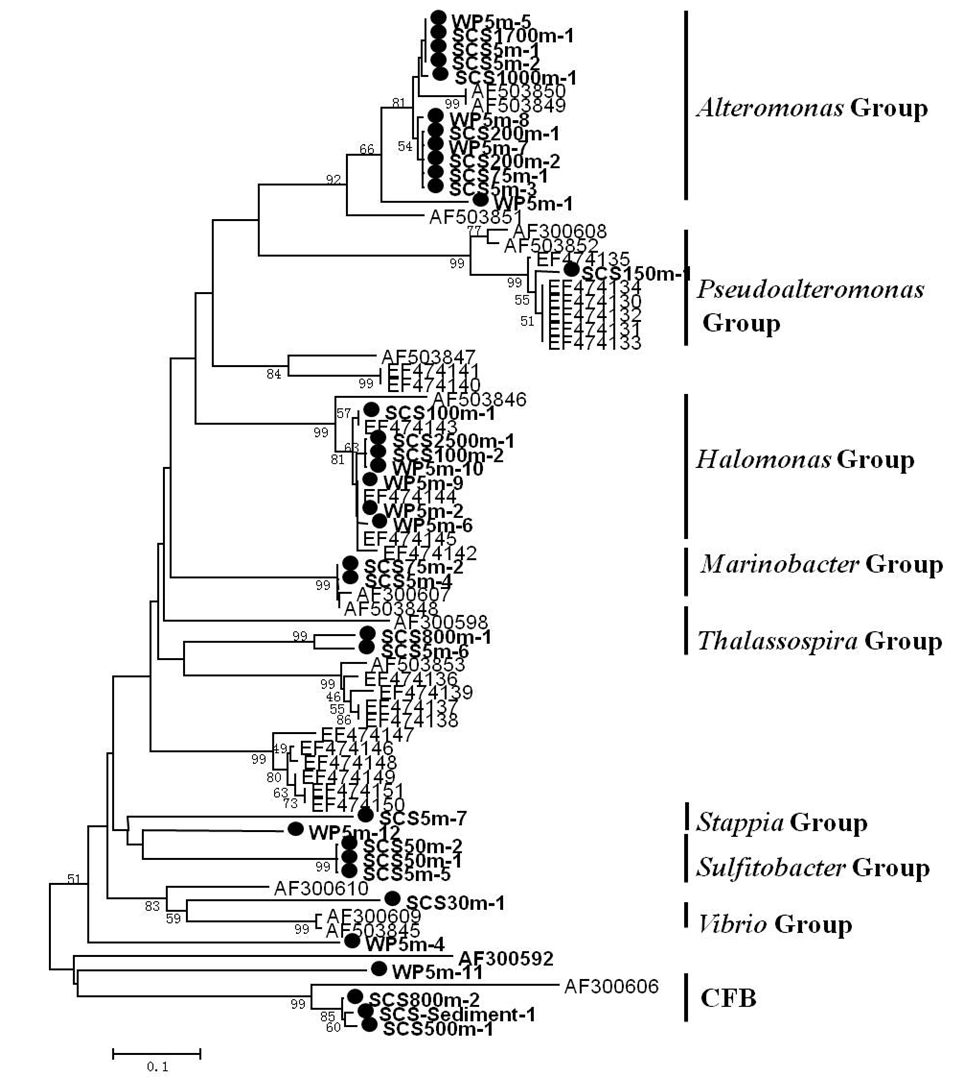

Supplement: S3 Fig — (TIF) [file pone.0117473.s003.tif]
